# Contribution of uniparental disomy to fetal growth restriction: a whole-exome sequencing series in a prenatal setting

**DOI:** 10.1038/s41598-023-50584-5

**Published:** 2024-01-02

**Authors:** Mengmeng Li, Na Hao, Yulin Jiang, Huili Xue, Yifang Dai, Mingming Wang, Junjie Bai, Yan Lv, Qingwei Qi, Xiya Zhou

**Affiliations:** 1 National Clinical Research Centre for Obstetric & Gynecologic Diseases, Department of Obstetrics and Gynecology, Peking Union Medical College Hospital, Chinese Academy of Medical Sciences and Peking Union Medical College, Beijing, 100730 China; 2 Medical Genetic Diagnosis and Therapy Center, Fujian Key Laboratory for Prenatal Diagnosis and Birth Defect, Fujian Maternity and Child Health Hospital College of Clinical Medicine for Obstetrics & Gynecology and Pediatrics, Fujian Medical University, Fujian, 350001 China; 3 GenoDecode (Beijing) Co. Ltd., Beijing, 101160 China; 4 Be Creative Lab (Beijing) Co. Ltd., Beijing, 100176 China

**Keywords:** Cytogenetics, Genomics

## Abstract

Fetal growth restriction (FGR), a leading cause of perinatal morbidity and mortality, is caused by fetal, maternal, and placental factors. Uniparental disomy (UPD) is a rare condition that leads to imprinting effects, low-level mosaic aneuploidies and homozygosity for pathogenic variants. In the present study, UPD events were detected in 5 women with FGR by trio exome sequencing (trio-WES) of a cohort of 150 FGR cases. Furthermore, noninvasive prenatal testing results of the 5 patients revealed a high risk of rare autosomal trisomy. Trio-WES showed no copy-number variations (CNVs) or nondisease-causing mutations associated with FGR. Among the 5 women with FGR, two showed gene imprinting, and two exhibited confined placental mosaicism (CPM) by copy number variant sequencing (CNV-seq). The present study showed that in FGR patients with UPD, the detection of imprinted genes and CPM could enhance the genetic diagnosis of FGR.

## Introduction

Fetal growth restriction (FGR) is defined as a condition in which the fetus fails to attain the genetic growth potential^1^. The incidence of FGR is approximately 3–7% of all pregnancies, and it is the second most common cause of perinatal mortality^2,3^. FGR also increases the risk of a variety of long-term adverse outcomes^4^. FGR has multifactorial etiologies, including placental, maternal and fetal factors. Guidelines and consensus recommend detailed ultrasound, genetic counseling and prenatal diagnosis when FGR is diagnosed^5,6^.

Among the genetic etiologies of FGR, uniparental disomy (UPD) might result in abnormal placental function due to its effects on the diploid cell line^7,8^ and therefore affect fetal growth. Although chromosomal microarray analysis (CMA) has been widely used in the prenatal diagnosis of FGR and UPD events may be detected ^9–11^, trio whole-exome sequencing (trio-WES) could detect the origin of UPD and more UPD events in prior normal CMA results^12^.

To investigate how UPD is related to FGR, we retrospectively analyzed FGR patients who underwent prenatal diagnosis with trio-WES.

## Results

From January 2020 to December 2022, 150 FGR cases (6.48%) were identified in 2314 trio-WES carried out in Be Creative Lab (Beijing). Among them, 9 LOH cases were noticed, and five UPD events were detected, including 3 segmental iUPD (isodisomy) with hUPD (heterodisomy) events on chromosomes 2, 6, and 15 and 2 iUPD events on chromosomes 6 and 15 (Table 1). The incidence of UPD in this FGR cohort was 3.33% (5/150).

**Table 1:** Variants in FGR cases with UPD detected by NIPT, CMA, WES, and CNV-seq.

Patient	Chr	Type of UPD	Parental origin	NIPT	WES clinical significance	CMA	Placenta CNV-seq/CMA	Clinical features
P1	15	Segmental iUPD with hUPD	Maternal	High risk of trisomy 15	Prader-Willi syndrome	arr[hg19] 15q21.3q26.1(51,970,001_89370001) × 2 hmz	NA	Intrauterine growth retardation、Fetal distress
P2	15	iUPD	Maternal	High risk of trisomy 15	Prader-Willi syndrome	arr[hg19]15q11.2q26.3(22,817,871_102,397,317) × 2 hmz	seq[hg19] dup(15) × 2–3	Intrauterine growth retardation
P3	6	iUPD	Maternal	High risk of trisomy 6	NM_014780.5(*CUL7*):c.509 T > G(p.Leu170Trp) (Uncertain significance)	arr(6) × 2 hmz	seq[hg19] dup(6) × 2 ~ 3	Intrauterine growth retardation
P4	6	Segmental iUPD with hUPD	Maternal	High risk of trisomy 6	Uncertain	arr[hg19] 6p25.3p23(203,878_13,411,320) × 2 hmz, 6p21.1p11.1(41,305,454_58,726,706) × 2 hmz, 6q11.1q14.1(61,972,918_75,972,465) × 2 hmz, 6q22.31q25.1(123,041,062_149,830,858) × 2 hmz	seq[hg19] (1–22) × 2,(XN) × 1	Intrauterine growth retardation, Fetal distress, Oligohydramnios
P5	2	Segmental iUPD with hUPD	Maternal	High risk of Trisomy 2	Uncertain	arr[hg19] 2p25.3p24.3(50,814_13,311,915) × 2 hmz, 2p21p11.2(45,974,85_87,053,152) × 2 hmz, 2q11.1q12.3(95,550,958_109,626,929) × 2 hmz 2q32.3q36.3(192,341,274_230,205,775) × 2 hmz	seq[hg19] dup(2) × 3	Oligohydramnios, Abnormality of calvarial morphology, Stillbirth

NA: not available.

The indications for prenatal diagnosis in the five cases were all high risk in NIPT, involving chromosomes 2, 6 and 15 (Table 1). After amniocentesis, karyotyping and CMA were carried out, and all had hmz (homozygous). Thus, trio-WES was undertaken; thereafter, all 5 fetuses presented early FGR.

**Figure 1:**
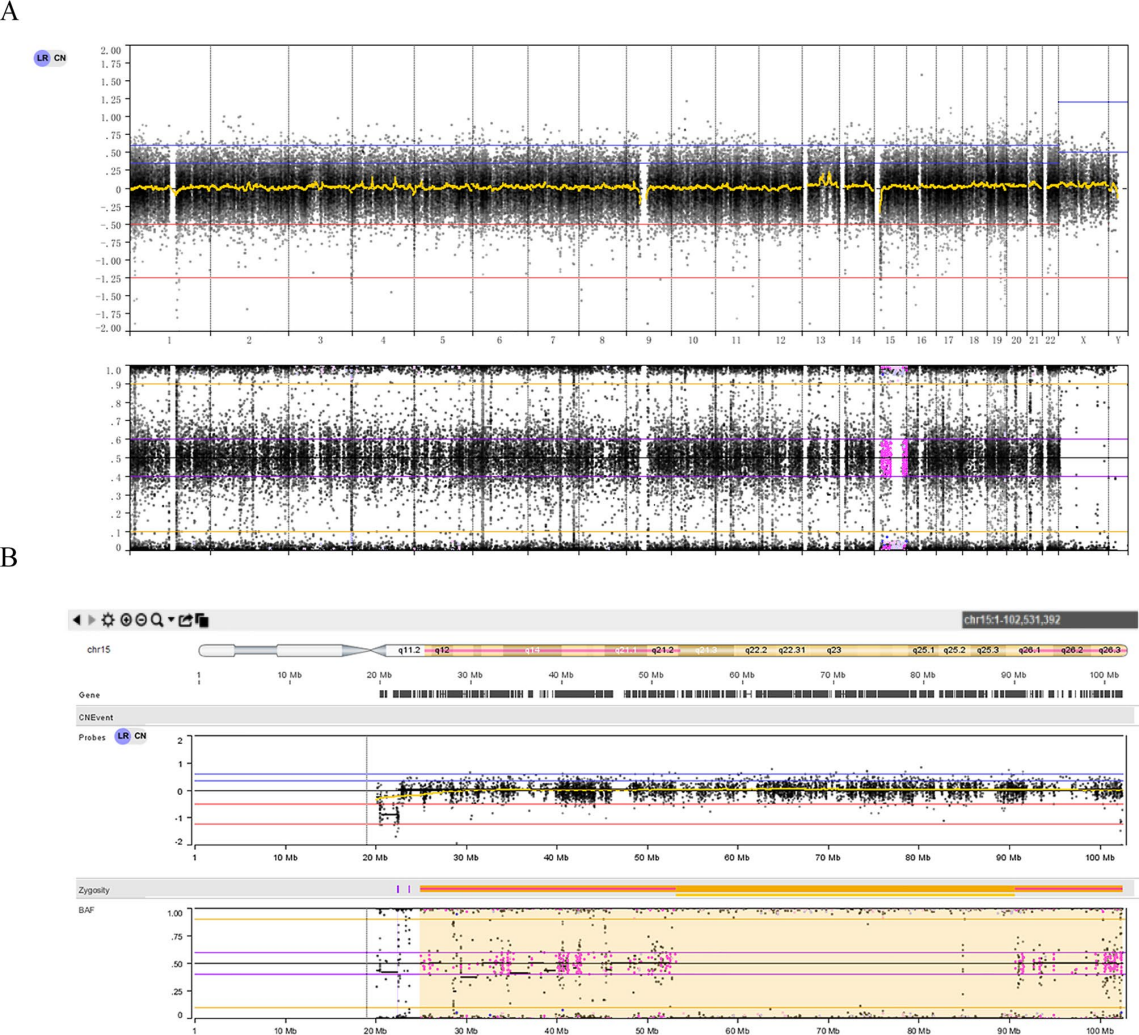
Genetic diagnosis of patient 1. (**A**) Trio-WES result of the normal CNV type. (**B**) Trio-WES result of chr15 showing segmental iUPD with hUPD.

**Figure 2:**
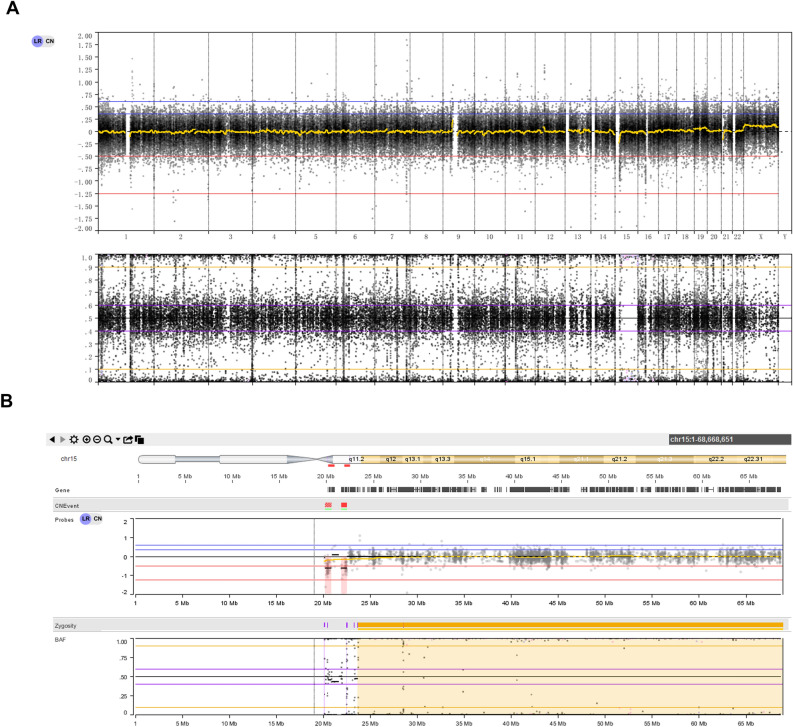
Genetic diagnosis of patient 2. (**A**) Trio-WES result of the normal CNV type. (**B**) Trio-WES result of chr15 showing segmental iUPD.

Patient 1 and patient 2 underwent NIPT and showed a high risk of trisomy 15. CMA detected a 37.4-Mb ROH at 15q21.3q26.1 (hg19:51,970,001_89,370,001) in patient 1 and whole chromosomal iUPD of 15 in patient 2. After genetic counseling, trio-WES was performed, and the results indicated segmental iUPD with hUPD of chromosome 15 in patient 1 (Fig. 1), while whole iUPD of chromosome 15 was confirmed in patient 2 (Fig. 2). The results revealed that only the maternal allele was present for both cases, which could cause Prader-Willi syndrome. FGR was detected in the prenatal ultrasonography. FGR was mainly due to gene imprinting that affected the growth of the fetuses. Trio-WES did not detect any pathogenic/likely pathogenic recessive variants associated with the clinical features on chromosome 15.

**Figure 3:**
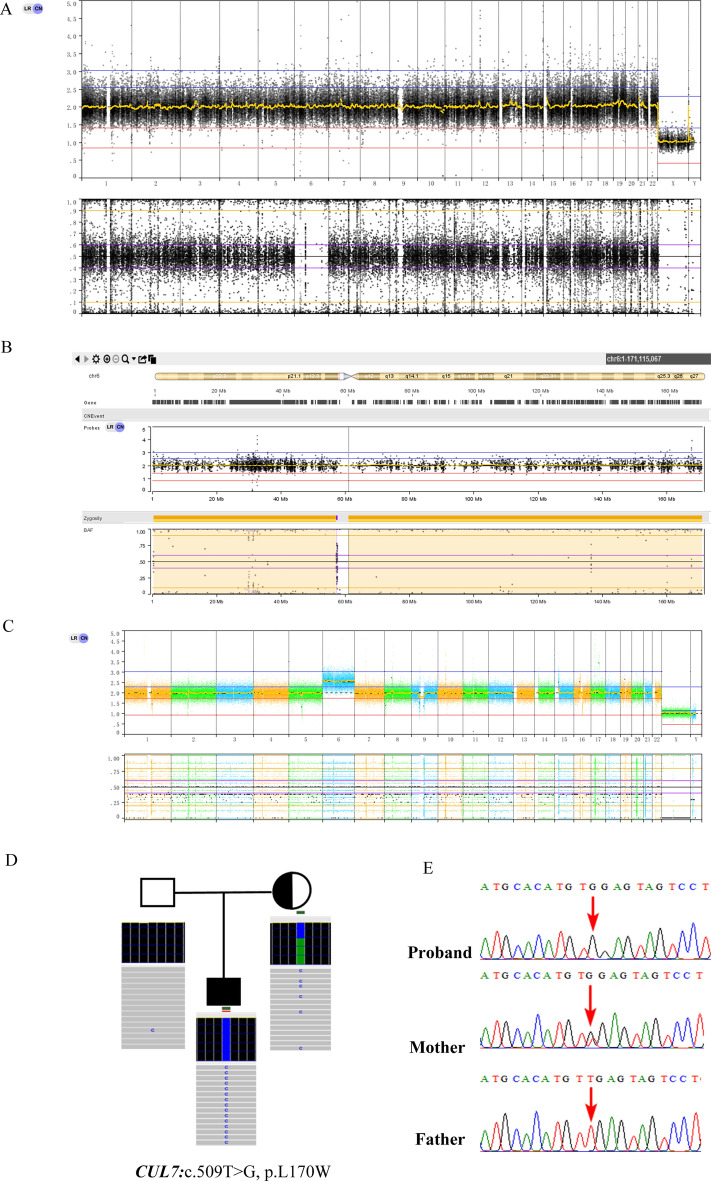
Genetic diagnosis of Patient 3. (**A**) Trio-WES result of the normal CNV type. (**B**) Trio-WES result of chr6 with segmental iUPD. (**C**) CNV-seq results of the placenta with suspected CPM involving trisomy 6. (**D**) Trio-WES result of homozygous *CUL7* c.509T > G(p.Leu170Trp) mutation. (**E**) Confirmation of homozygous *CUL7* c.509T > G(p.Leu170Trp) by Sanger sequencing.

Patient 3 received a prenatal diagnosis because NIPT indicated a high risk of trisomy 6. CMA showed no clinically significant copy number variations. However, it revealed LOH across the entire chromosome 6. After genetic counseling, trio-WES was performed. Complete maternal iUPD for chromosome 6 was confirmed. Trio-WES also identified a homozygous *CUL7* c.509 T > G (p.Leu170Trp) mutation in the fetus, which was classified as a variant of unknown significance (VOUS). The mutation was confirmed by Sanger sequencing and was inherited from the mother; the father did not carry the mutation (Fig. 3). Patient 3 was diagnosed with CPM based on the result of CNV-Seq of the placenta.

**Figure 4:**
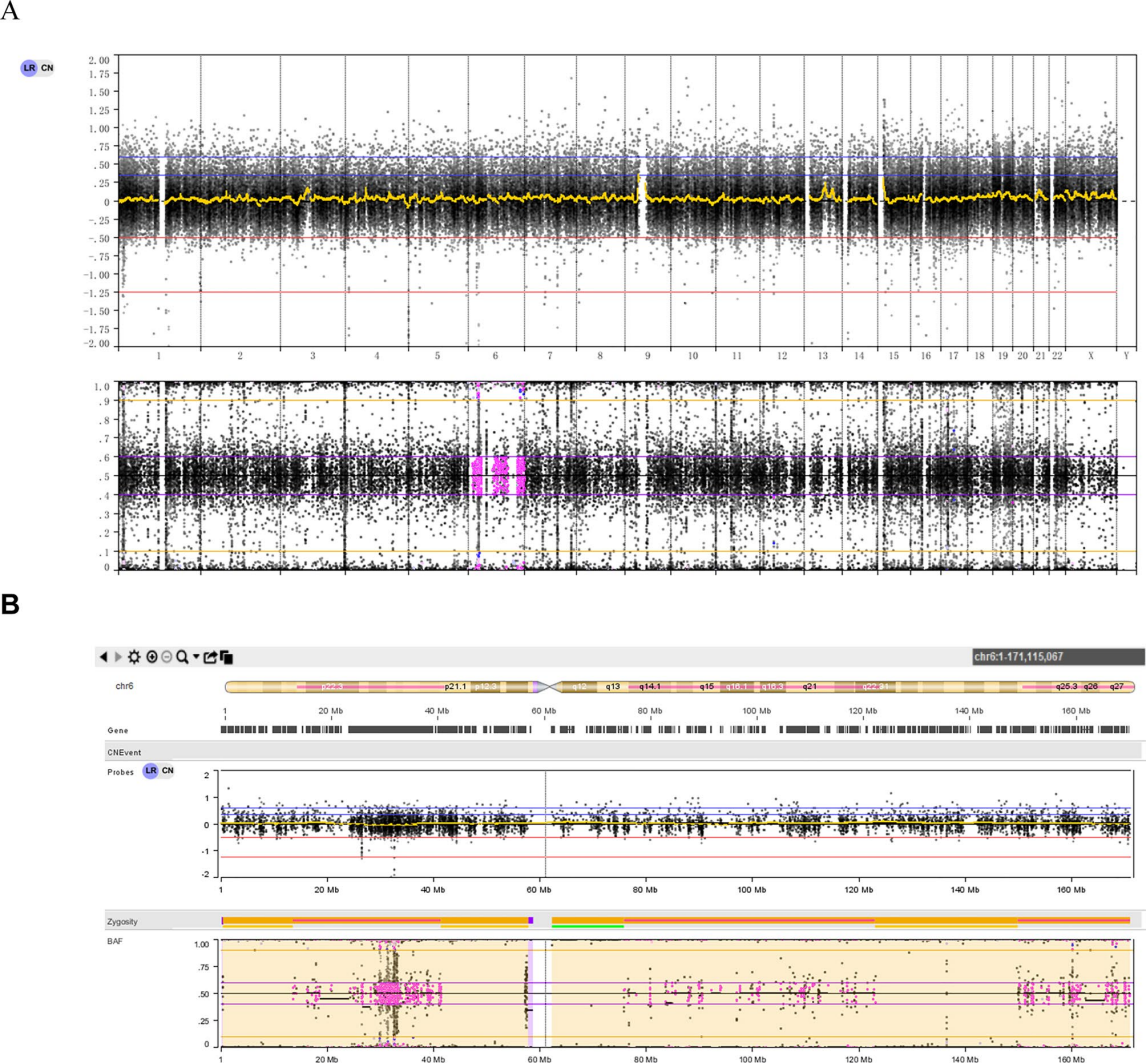
Genetic diagnosis of Patient 4. (**A**) Trio-WES result of the normal CNV type. (**B**) Trio-WES result of chr6 showing segmental iUPD with hUPD.

Patient 4 showed a high risk of trisomy 6 by NIPT. CMA detected LOH fragments on chromosome 6. Trio-WES showed maternal segmental iUPD with hUPD (Fig. 4). The prenatal ultrasound revealed FGR and oligohydramnios. No pathogenic/likely pathogenic recessive variants were detected. Placental CNV-seq revealed no abnormalities.

**Figure 5:**
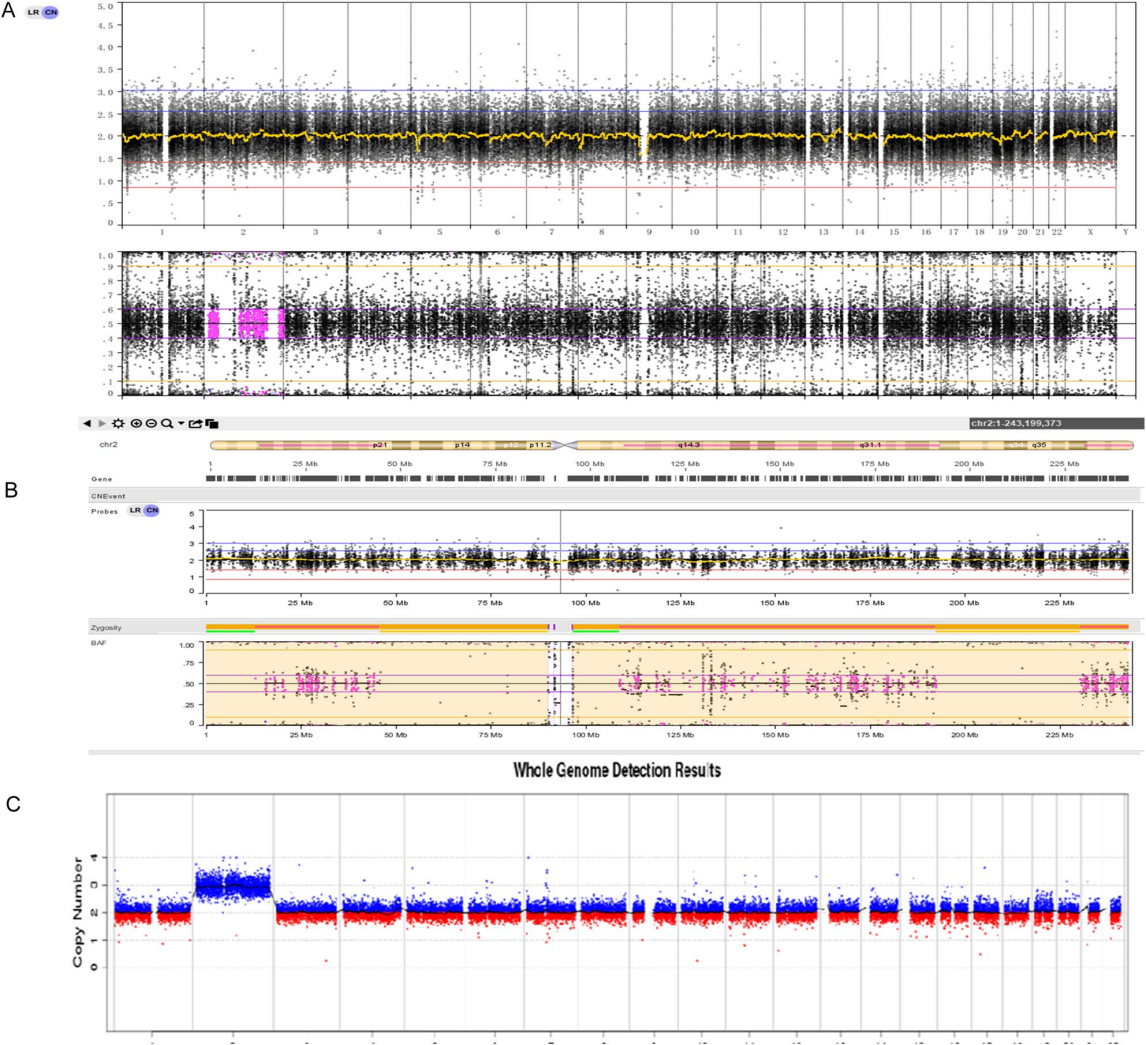
Genetic diagnosis of Patient 5. (**A**) Trio-WES result of the normal CNV type. (**B**) Trio-WES result of chr2 showing segmental iUPD with hUPD. (**C**) CNV-seq results of the placenta with suspected CPM involving trisomy 2.

Patient 5 underwent NIPT and showed a high risk of trisomy 2. The fetus had a normal karyotype, yet CMA revealed several LOH fragments on chromosome 2 (Fig. 5). No disease-related imprinting genes were located on chromosome 2. Trio-WES was then performed. No homozygous mutations of any known recessive pathogenic genes for inherited disorders were detected on chromosome 2. However, the LOH analysis revealed that chromosome 2 was maternal segmental iUPD with hUPD (Fig. 5). The fetus showed severe FGR, and intrauterine fetal death occurred at the 23^rd^ week of gestation. The placenta was proven to be a complete trisomy 2 by CNV-Seq.

## Discussion

FGR is a condition in which the fetus fails to reach its genetic growth potential due to a variety of factors. The most common genetic etiology of FGR involves aneuploidy and CNV^11,13^. It has been reported that at least 15% to 20% of FGR cases are caused by chromosomal abnormalities^14,15^. Confined placental mosaicism (CPM) was also reported to be the genetic etiology of FGR, and FGR was reported in 71.7% of CPM cases ^8^.

UPD refers to a condition in which both copies of a chromosome pair are inherited from one parent^16^. UPD has been reported in nearly all chromosomes^17^. The major mechanisms of UPD are trisomic rescue and monosomic rescue^18^. Trisomic rescue is a rescue of the aneuploidy by loss of the third chromosome which accounts for one-third of UPD cases, whereas monosomic rescue is a rescue of aneuploidy by duplication of a monosomic chromosome^18^. Several problems are associated with UPD, e.g., an imprinted chromosome is involved, homozygosity of autosomal recessive mutations is available, or CPM occurs in the placenta^19^. The clinical outcome of UPD depends on its origin and the chromosome involved. Some UPD events were reported to be associated with FGR, including paternal UPD6 and UPD15 and maternal UPD 6, UPD 7, UPD11, UPD14, UPD15 and UPD20^7,8,20–24^.

CMA has been considered the first-tier test for prenatal diagnosis of FGR^25^. Although whole chromosome iUPD can be detected by CMA ^10^, routine analysis cannot identify whole chromosome hUPD, and the parental origin cannot be confirmed without parental samples. Thus, approximately one-third of all cases of molecularly confirmed UPD were not detectable by CMA^18,26,27^. In the last decade, trio-WES has been increasingly used in prenatal settings^28,29^. Although it has not been the first tier choice in prenatal diagnosis, trio-WES can detect all types of UPD, including iUPD, hUPD, mixed UPD and segmental UPD^12^. Kevin Yauy et al. (2020) identified 10 UPD events in 4912 trio-WES^30^. Julie Scuffins et al. (2021) detected 112 UPD events in 32,067 trio-WES and 13 UPD events in prior normal CMA results^12^. Currently, there is no sufficient evidence on the diagnosis yield of trio-WES in FGR fetuses. Several recent meta-analyses have demonstrated an added diagnostic yield of 1.8–68% for prenatal WES, with the yield largely depending on the inclusion criteria and organ system affected^31–33^. When FGR is associated with multisystem structural abnormalities, trio WES should be recommended and offered^34,35^.

In the current research, we identified 5 UPD cases by trio-WES from a cohort of 150 pregnancies complicated by FGR, and the type of UPD was confirmed. The indications of prenatal diagnosis were all abnormal NIPT results, and trio-WES showed maternal UPD 2, UPD 6 and UPD 15. UPD in patient 1 and patient 2 lay in the 15q11-13 imprinted region on the maternal allele, which resulted in Prader-Willi syndrome characterized by growth deficit in utero. Patient 3 and patient 4 had UPD 6, which was reported to cause FGR. However, patient 5 had maternal UPD 2, which has not been reported to cause FGR.

CPM is associated with FGR when chromosomes 2, 3, 7, 13, 15, 16, or 22 are involved^36,37^. In our series, CPM was suspected because all cases were NIPT positive and had maternal UPD. As there was no imprinting region on chromosome 2 and CPM was confirmed in the placenta of patient 5, it was speculated that the etiology of FGR in patient 5 was CPM.

Here, we illustrated a retrospective study of UPD events detected by trio-WES in FGR patients. Although it is a small series, it revealed that for FGR pregnancies with abnormal results in NIPT, trio-WES might be suggested to detect fetal SNP mutations, CNV and UPD events simultaneously. In addition to CPM, UPD is a genetic etiology of FGR as well.

## Methods

### Study design

The prenatal trio-WES database between 2020 and 2022 in Be Creative Lab (Beijing) was searched by “singleton pregnancy” and phenotype “fetal growth restriction”. FGR was defined as the estimated fetal weight under the 10th percentile for gestational age^6^. Fetuses with major anomalies or chromosomal abnormalities were excluded.

In all FGR cases with trio-WES results, cases of loss of heterozygosity (LOH) were noticed. Among them, UPD was confirmed using B allele frequency (BAF). Prenatal diagnosis profiles and pregnancy outcomes were analyzed.

### Whole-exome sequencing

Genomic DNA was extracted from peripheral blood samples and uncultured amniotic fluid samples obtained from pregnant women and their fetuses by using the QIAamp DNA Blood Mini Kit in accordance with the manufacturer’s instructions. A DNA library was prepared using Illumina protocols and sequenced on the NovaSeq 6000 platform (Illumina, USA) in accordance with the manufacturer’s instructions. All exon regions and 20 bp of exon-flanking intron regions were captured for sequencing.

Quality control of the WES data was performed with fastq. Mapping (bwa) and variant calling (GATK, SAMtools, and Freebayes) were streamlined with the SeqMule pipeline by using the GRCh37 genome^38^ Genetic variants were annotated by ANNOVAR. All identified variants with a minor allele frequency of < 0.05 in the control population database of the gnomAD (http://gnomad.broadinstitute.org/) were evaluated for pathogenicity. The mutations were classified according to the standards and guidelines of the American College of Medical Genetics and Genomics (ACMG). Variants found in fetuses were then compared with those in their parents. All candidate pathogenic mutations were confirmed by Sanger sequencing.

CNV and UPD were detected using NxClinical software and BAM files as input with the following parameters: significance threshold = 1.0E-6, high gain (4 + :2) = 0.6, gain (3:2) = 0.18, loss (1:2) = −0.18, large loss (0:2) =  −1.0, homozygous frequency threshold = 0.97, homozygous value threshold = 0.8, heterozygous imbalance threshold = 0.4, minimum loss of heterozygosity (LOH) length (kb) = 2000, and minimum single nucleotide polymorphism (SNP) probe density (probes/Mb) = 0. UPD was detected using “B Allele Frequency” (BAF) and the identity of the LOH that covers the entire chromosome. NxClinical was used to detect UPD within the trios to confirm the maternal or paternal origin of UPD by using informative BAF values from the proband and parent sample(s). The Database of Genomic Variants (DGV), the Database of Chromosome Imbalance and Phenotype in Humans Using Ensemble Resources (DECIPHER), the Clinical Genome Resource (ClinGen), and Online Mendelian Inheritance in Man (OMIM) were used to evaluate the mutations identified in this study.

### CMA analysis

Genomic DNA was extracted from uncultured amniotic fluid, villus, or cord blood samples using the QIAamp DNA Blood Mini Kit (Qiagen, Valencia, CA, USA). Next, 250 ng of the extracted genomic DNA was digested, ligated, amplified by PCR, labeled, and hybridized with the CytoScan 750 K Array (Affymetrix, Santa Clara, CA, USA) in accordance with the manufacturer’s protocol. After washing and staining, the arrays were scanned with the Affymetrix GeneChip Scanner 3000. The obtained data were analyzed with Chromosome Analysis Suite v4.2 software. The GRCh37 genome was used to annotate CNVs. The thresholds were as follows: > 200 kb for gains, > 100 kb for losses, and > 10 Mb for region of homozygosity (ROH).

### CNV-Seq

Genomic DNA was extracted from the samples for library construction by using rapid PCR-free library construction technology. Next, 10 ng genomic DNA was randomly fragmented using a nebulizer. A DNA library was prepared. The quality of the library was assessed, and the library was then sequenced on the NovaSeq 6000 platform (Illumina). The data were analyzed with NxClinical software.

### Sanger sequencing

Candidate variants were confirmed by Sanger sequencing. The amplified fragments were sequenced on an ABI 3730 genetic analyzer (Applied Biosystems, USA).

### Ethical approval and consent to participate

This study was carried out following the Helsinki Declaration's ethical guidelines. Informed consent was obtained from parents. The ethical committees of the Peking Union Medical College Hospital (I-23PJ333) approved the study protocol.

## Data Availability

The datasets generated and/or analyzed during the current study are not publicly available in order to comply with hospital and IRB policy. According to the consent form, sequencing data can not be accessed without patient's permission. But they are available from the corresponding author on reasonable request.

## References

[1] Chew, L. C. & Verma, R. P. Fetal Growth Restriction. In: *StatPearls.* Treasure Island (FL) ineligible companies. Disclosure: Rita Verma declares no relevant financial relationships with ineligible companies. (2023).

[2] Nardozza LM, Araujo Junior E, Barbosa MM, Caetano AC, Lee DJ, Moron AF (2012). Fetal growth restriction: current knowledge to the general Obs/Gyn. Arch. Gynecol. Obstet..

[3] Romo A, Carceller R, Tobajas J (2009). Intrauterine growth retardation (IUGR): epidemiology and etiology. Pediatr. Endocrinol. Rev..

[4] Crispi F, Miranda J, Gratacos E (2018). Long-term cardiovascular consequences of fetal growth restriction: biology, clinical implications, and opportunities for prevention of adult disease. Am. J. Obstet. Gynecol..

[5] Fetal Growth Restriction (2021). ACOG Practice Bulletin, Number 227. Obstet. Gynecol..

[6] Sun L, Hu Y, Qi H, Fetal Medicine Subgroup CSoPM, Chinese Medical Association, Maternal-Fetal Medicine Committee CSoO, Gynecology CMA (2022). A summary of chinese expert consensus on fetal growth restriction (An update on the 2019 version). Maternal-Fetal Med..

[7] Maulik D (2006). Fetal growth restriction: the etiology. Clin. Obstet. Gynecol..

[8] Redaelli S, Sala E, Roncaglia N, Colombo C, Crosti F, Villa N, Tagliabue P, Cappellini A, Dalpra L (2005). Severe intrauterine growth restriction and trisomy 15 confined placental mosaicism: a case report and review of literature. Prenat. Diagn..

[9] An G, Lin Y, Xu LP, Huang HL, Liu SP, Yu YH, Yang F (2018). Application of chromosomal microarray to investigate genetic causes of isolated fetal growth restriction. Mol. Cytogenet..

[10] Wu X, He S, Li Y, Guo D, Chen X, Liang B, Wang M, Huang H, Xu L (2023). Fetal genetic findings by chromosomal microarray analysis and karyotyping for fetal growth restriction without structural malformations at a territory referral center: 10-year experience. BMC Pregnancy Childbirth.

[11] Chen Y, Xie Y, Jiang Y, Luo Q, Shi L, Zeng S, Zhuang J, Lyu G (2021). The genetic etiology diagnosis of fetal growth restriction using single-nucleotide polymorphism-based chromosomal microarray analysis. Front. Pediatr..

[12] Scuffins J, Keller-Ramey J, Dyer L, Douglas G, Torene R, Gainullin V, Juusola J, Meck J, Retterer K (2021). Uniparental disomy in a population of 32,067 clinical exome trios. Genet. Med..

[13] Bahado-Singh RO, Lynch L, Deren O, Morroti R, Copel JA, Mahoney MJ, Williams J (1997). First-trimester growth restriction and fetal aneuploidy: the effect of type of aneuploidy and gestational age. Am. J. Obstet. Gynecol..

[14] Zhu H, Lin S, Huang L, He Z, Huang X, Zhou Y, Fang Q, Luo Y (2016). Application of chromosomal microarray analysis in prenatal diagnosis of fetal growth restriction. Prenat. Diagn..

[15] Borrell A, Grande M, Meler E, Sabria J, Mazarico E, Munoz A, Rodriguez-Revenga L, Badenas C, Figueras F (2017). Genomic microarray in fetuses with early growth restriction: a multicenter study. Fetal. Diagn. Ther..

[16] Robinson WP (2000). Mechanisms leading to uniparental disomy and their clinical consequences. Bioessays.

[17] Nakka P, Pattillo Smith S, O’Donnell-Luria AH, McManus KF, Meresearch T, Mountain JL, Ramachandran S, Sathirapongsasuti JF (2019). Characterization of prevalence and health consequences of uniparental disomy in four million individuals from the general population. Am. J. Hum. Genet..

[18] Del Gaudio D, Shinawi M, Astbury C, Tayeh MK, Deak KL, Raca G, Committee ALQA (2020). Diagnostic testing for uniparental disomy: a points to consider statement from the American College of medical genetics and genomics (ACMG). Genet. Med..

[19] Kotzot D (2008). Prenatal testing for uniparental disomy: indications and clinical relevance. Ultrasound Obstet. Gynecol..

[20] Lazier J, Martin N, Stavropoulos JD, Chitayat D (2016). Maternal uniparental disomy for chromosome 6 in a patient with IUGR, ambiguous genitalia, and persistent mullerian structures. Am. J. Med. Genet. A.

[21] Eggermann T (2010). Russell-Silver syndrome. Am. J. Med. Genet. C Semin. Med. Genet..

[22] Wang LQ, Fernandez-Boyano I, Robinson WP (2022). Genetic variation in placental insufficiency: What have we learned over time?. Front. Cell Dev. Biol..

[23] Sutton VR, Shaffer LG (2000). Search for imprinted regions on chromosome 14: comparison of maternal and paternal UPD cases with cases of chromosome 14 deletion. Am. J. Med. Genet..

[24] Mulchandani S, Bhoj EJ, Luo M, Powell-Hamilton N, Jenny K, Gripp KW, Elbracht M, Eggermann T, Turner CL, Temple IK (2016). Maternal uniparental disomy of chromosome 20: a novel imprinting disorder of growth failure. Genet. Med..

[25] Hay SB, Sahoo T, Travis MK, Hovanes K, Dzidic N, Doherty C, Strecker MN (2018). ACOG and SMFM guidelines for prenatal diagnosis: Is karyotyping really sufficient?. Prenat. Diagn..

[26] Kearney HM, Kearney JB, Conlin LK (2011). Diagnostic implications of excessive homozygosity detected by SNP-based microarrays: consanguinity, uniparental disomy, and recessive single-gene mutations. Clin. Lab. Med..

[27] Hoppman N, Rumilla K, Lauer E, Kearney H, Thorland E (2018). Patterns of homozygosity in patients with uniparental disomy: detection rate and suggested reporting thresholds for SNP microarrays. Genet. Med..

[28] Lord J, McMullan DJ, Eberhardt RY, Rinck G, Hamilton SJ, Quinlan-Jones E, Prigmore E, Keelagher R, Best SK, Carey GK (2019). Prenatal exome sequencing analysis in fetal structural anomalies detected by ultrasonography (PAGE): a cohort study. Lancet.

[29] Petrovski S, Aggarwal V, Giordano JL, Stosic M, Wou K, Bier L, Spiegel E, Brennan K, Stong N, Jobanputra V (2019). Whole-exome sequencing in the evaluation of fetal structural anomalies: a prospective cohort study. Lancet.

[30] Yauy K, de Leeuw N, Yntema HG, Pfundt R, Gilissen C (2020). Accurate detection of clinically relevant uniparental disomy from exome sequencing data. Genet. Med..

[31] Mellis R, Oprych K, Scotchman E, Hill M, Chitty LS (2022). Diagnostic yield of exome sequencing for prenatal diagnosis of fetal structural anomalies: A systematic review and meta-analysis. Prenat. Diagn..

[32] Pauta M, Martinez-Portilla RJ, Borrell A (2022). Diagnostic yield of exome sequencing in fetuses with multisystem malformations: systematic review and meta-analysis. Ultrasound Obstet. Gynecol..

[33] Pauta M, Martinez-Portilla RJ, Borrell A (2021). Prenatal exome sequencing in recurrent fetal structural anomalies: systematic review and meta-analysis. J. Clin. Med..

[34] Mone F, Mellis R, Gabriel H, Baptiste C, Giordano J, Wapner R, Chitty LS (2023). Should we offer prenatal exome sequencing for intrauterine growth restriction or short long bones? A systematic review and meta-analysis. Am. J. Obstet. Gynecol..

[35] Shi D, Cai L, Sun L (2022). Genetics etiologies associated with fetal growth restriction. Maternal-Fetal. Med..

[36] Eggenhuizen GM, Go A, Koster MPH, Baart EB, Galjaard RJ (2021). Confined placental mosaicism and the association with pregnancy outcome and fetal growth: a review of the literature. Hum. Reprod. Update.

[37] Wilkins-Haug L, Quade B, Morton CC (2006). Confined placental mosaicism as a risk factor among newborns with fetal growth restriction. Prenat. Diagn..

[38] Guo Y, Ding X, Shen Y, Lyon GJ, Wang K (2015). SeqMule: automated pipeline for analysis of human exome/genome sequencing data. Sci. Rep..

